# Characterization of Băbească Neagră Grape Pomace and Incorporation into Jelly Candy: Evaluation of Phytochemical, Sensory, and Textural Properties

**DOI:** 10.3390/foods13010098

**Published:** 2023-12-27

**Authors:** Mariana Spinei, Mircea Oroian

**Affiliations:** 1 Integrated Center for Research, Development and Innovation in Advanced Materials, Nanotechnologies, and Distributed Systems for Fabrication and Control (MANSiD), “Ştefan cel Mare” University of Suceava, 13th University Street, 720229 Suceava, Romania; 2 Faculty of Food Engineering, “Ştefan cel Mare” University of Suceava, 13th University Street, 720229 Suceava, Romania; m.oroian@fia.usv.ro

**Keywords:** grape pomace, fortification, jelly candy, valorization

## Abstract

The influence of particle size intervals (<125 μm, ≥125–<200 μm, and ≥200–<300 μm) of grape pomace (*Vitis vinifera* var. Băbească Neagră) was analyzed in terms of the proximate composition, functional properties, and physicochemical parameters. The aim was to study the effect of the formulation variables (extract from grape pomace with different particle size intervals and gelatin doses—7, 8.5, and 10 g) on the mechanical properties (hardness, cohesiveness, adhesiveness, springiness, and gumminess), color, and sensorial, microbiological, and phytochemical parameters. The jelly candy formulated with grape pomace extract (<125 μm) and 7 g of gelatin showed the highest total phenolic content (156 mg GAE/g) and antioxidant activity (65.8% inhibition), while grape pomace jellies with a particle size of ≥125–<200 μm and different concentration of gelatin presented the greatest sensory acceptance in terms of sweetness, taste, odor, elasticity, color, and overall acceptability of the resulting jelly. The concluding observation was supported by the microbiological analysis, which also showed that there is no growth in jelly samples except the jelly candies prepared with a ≥200–<300 μm particle size interval of grape pomace extract.

## 1. Introduction

The grape has been one of the most planted fruits for a long time, consumed in a variety of ways [1,2], and it is classified as a berry produced by the woody vines of the plant genus *Vitis* [2]. The grapevine, native to Europe and the Mediterranean region, relates to the species *Vitis vinifera*, which consists of approximately 10,000 distinct varieties [3]. Depending on their uses, cultivated grapes can be divided into three different kinds, i.e., wine, table, and dried grapes [2,4]. Table grapes are utilized for the production of jelly, jam, juice, juice concentrate, vinegar, and other products, although grapes can also be dehydrated into raisins [5].

Wine grapes have a significant part in the global economy [6]; in 2022, the world vineyard surface area was estimated to be 7.3 mha [7]. Looking at the European Union (EU) member states, Romania had about 188 kha of vineyard surface in 2022 with 3.9 mhL of the world wine production, excluding musts and juices [7]. The major by-products that are generated after the maceration process are grape pomace, grape seeds, yeast, etc. [1]. Grape pomace or grape marc comprises three different constituents: skins, seeds, and stalks remaining after various stages of wine production, such as crushing, draining, and pressing processes [8]. One of the main destinations of the grape pomace is distillation in order to obtain alcohol [8]. In addition, this by-product is commonly used as fertilizer, bio-fuel, bio-based material, or fodder, but can also be used for the extraction of different compounds, such as phenols, tocopherols, tannins, oil, etc. [1]. Therefore, there is a growing interest in grape pomace as a value-added compound in food products. In recent times, grape pomace has been used as a fortifying agent for a lot of food products, e.g., plant foodstuffs (bread, cookies, extruded cereals, muffins, biscuits, pancakes, noodles, tomato puree, and pasta), meat and fish products (pork sausages, pork burgers, chicken meat, beef frankfurters, minced fish muscles, pork loin marinade, and salmon burgers), and dairy products (cheese, fermented milk, yogurt, and ice cream) [9]. Confectionary products (e.g., nougat, hard and soft jelly candies) are attracting growing interest considering their high organoleptic indicators and low prices in comparison with other types of candy [10]. Jelly candy is consumed in great amounts, especially by children; this consumption can negatively influence health because of coloring agents, contaminants obtained during heat treatment, and artificial flavoring [10].

Romania has a significant range of indigenous varieties (*Vitis vinifera* L.), widely distributed throughout the country, e.g., Fetească Neagră, Băbească Neagră, Busuioacă Neagră, Busuioacă de Bohotin, Fetească Albă, Grasă de Cotnari, Tămâioasă Românească, Galbenă de Odobești, and Riesling de Banat [11]. Băbească Neagră (also Rară Neagră) is a dark-skinned grape variety native to Romania and the Republic of Moldova; the grapes are medium to large, cylindrical–conical with medium-sized and spherical berries which easily accumulate significant amounts of sugar (210–220 g/L) [12]. According to the operational data portal of the Ministry of Agriculture and Rural Development (MARD), Băbească Neagră occupied 2613 ha of the main noble wine grape varieties registered in cultivation in 2020 [13]. Waste minimization, sustainable food production, and valorization of by-products are important to prolong the shelf life of food products, produce new products, improve the nutritional value of products, and make products available out of season [14]. In the scientific literature, there are some studies on the valorization of grape-pomace (Kalecik Karasi cultivar) into soft candies, and their characterization from texture profile analysis, sensory analysis, color, opacity, and rheology testing point of view [15], but the study does not deal with proximate composition, FT-IR analysis, and SEM analysis of the pomace, nor with the phytochemical profile of the finished product. Moreover, the final product is different since soft candies are sweets in which sucrose esters can improve softness and comprise a blend of at least saccharide, vegetable oil, and emulsifier, while jelly candies (hard candies) are a broad category of gelatin-based chewable sweets. To our knowledge, there are no other studies on the valorization of Băbească Neagră grape pomace as a fortifying agent in jelly candies.

Consequently, the aim of this study was to improve knowledge about the fortification process in food products, mainly jellied products, to produce jelly candy, and to evaluate physicochemical (color parameters), phytochemical (total phenolic content and DPPH assay scavenging activity), textural (hardness, cohesiveness, adhesiveness, springiness, and gumminess), sensorial, and microbiological characteristics of the final product.

## 2. Materials and Methods

### 2.1. Plant Material and Jelly Candy Preparation

Grape pomace was collected by processing the Băbească Neagră variety (*Vitis vinifera* L.) 2019 harvest. The grape pomace was dried in an air-circulating oven Zhicheng ZRD-A5055 (Zhicheng, Shanghai, China) for 12 h at 50 °C. Then, dried pomace was powdered using a laboratory mill Perten Instruments LM 3310 (PerkinElmer Inc., Waltham, MA, USA) and sieved using a sieve shaker Retsch AS 200 Basic (Retsch GmbH, Haan, Germany) in order to obtain three different particle sizes: <125 μm, ≥125–<200 μm, and ≥200–<300 μm.

The different steps of the by-product-based jelly candy preparation process were:

1. Aqueous extract (*w*/*v*): 1 g of grape pomace with each particle size was mixed with 30 mL of water; the samples were homogenized with an Ultra-Turrax Homogenizer (Daigger Scientific Inc., Vernon Hills, IL, USA) for 5 min and heated for 1 h at 70 °C in a water bath Precisdig (J.P. Selecta, Barcelona, Spain). At the end of the treatment, the samples were centrifuged for 10 min at 5000 rpm; the supernatant was filtered and used for jelly candy formulation.
2. Gelatin hydration (*w*/*v*): 7, 8.5, and 10 g of gelatin (to make 100 g of jelly) were hydrated with water for 10 min at 40 °C.
3. Heat treatment and homogenization (*v*/*v*): the final mixture consisted of 12.5 g sweetener (stevia) and 210 mL of grape pomace extract for the products with 7, 8.5, and 10 g of gelatin, respectively (to make 100 g of jelly). These blends were heated for 5 min at 55 °C in a Thermomix device (Vorwerk, Wuppertal, Germany). Finally, the different formulations were established (F1–7, F1–8.5, F1–10, F2–7, F2–8.5, F2–10, F3–7, F3–8.5, and F3–10; F1—grape pomace extract with the particle size of <125 μm; F2—grape pomace extract with the particle size of ≥125–<200 μm; F3—grape pomace extract with the particle size of ≥200–<300 μm; 7—7 g of gelatin; 8.5—8.5 g of gelatin; 10—10 g of gelatin). The molding and maturation stages were performed at 5 °C for 24 h.

The information about the production of grape pomace jelly is presented in Table S1 of the Supplementary Materials.

### 2.2. Methods

#### 2.2.1. Characterization of Grape Pomace and Grape Pomace Aqueous Extract

Grape pomace of the Băbească Neagră variety (*Vitis vinifera* L.) was analyzed for proximate composition by AOAC methods for moisture content (g/100 g), crude fat (g/100 g), crude protein (g/100 g), ash (g/100 g), total dietary fiber (TDF, g/100 g), soluble dietary fiber (SDF, g/100 g), and insoluble dietary fiber (IDF, g/100 g) [16]. Other carbohydrates were determined according to the difference method by subtracting the other components from 100 (100 − (ash + protein + fat + moisture)).

For a better understanding of jelly candy composition, the phytochemical characteristics (total phenolic content and DPPH assay scavenging activity), moisture content (%), crude protein (g/100 mL), crude fat (g/100 mL), ash (g/100 mL), and total dietary fiber (g/100 mL) of grape pomace aqueous extract were analyzed according to the method described by Spinei and Oroian [17] and the AOAC methods [16]. Other carbohydrates were determined according to the difference method by subtracting the other components from 100 (100 − (ash + protein + fat + moisture)).

#### 2.2.2. Functional Properties of Grape Pomace

Water-holding capacity (WHC, g/g), swelling capacity (SC, mL/g), oil-binding capacity (OBC, g/g), and water solubility index (WSI, %) were determined according to methods previously described by Zhang et al. [18] with slight modifications detailed by Gao et al. [19].

#### 2.2.3. Color of Grape Pomace and Jelly Candy

The color of the grape pomace and jelly candy samples was analyzed in conformity with the method reported by Spinei and Oroian [20], using the granular material attachment CR-A50 (Konica Minolta, Inc., Tokyo, Japan). A glass Petri dish containing grape pomace and jelly candies was placed above the attachment.

#### 2.2.4. Phytochemical Characterization of Grape Pomace Extract

##### Extract Preparation

The extraction of the phytochemicals from the dried grape pomace was accomplished in accordance with the protocol reported by Spinei and Oroian [17] with slight modifications. Briefly, 1 g of grape pomace of the Băbească Neagră variety with different particle sizes, <125 μm, ≥125–<200 μm, and ≥200–<300 μm, was mixed with 25 mL of ethanol (70%, *w*/*v*), the samples were homogenized using an Ultra-Turrax Homogenizer (Daigger Scientific Inc., Vernon Hills, IL, USA) for 3 min, and heated for 30 min at 70 °C in a water bath Precisdig (J.P. Selecta, Barcelona, Spain). At the end of the treatment, the samples were centrifuged for 10 min at 5000 rpm. The supernatant was filtered and diluted 1:10 with ethanol 70% (*v*/*v*); then, it was described with regard to the total phenolic content (TPC), total flavonoid content (TFC), total monomeric anthocyanin content (TMA content), and antioxidant activity.

##### Bioactive Compounds and Antioxidant Activity of Grape Pomace Extract

The TPC was determined using the Folin–Ciocalteu method described by Spinei and Oroian [17]. The results were represented as mg of gallic acid equivalents per gram of grape pomace extract. The polyphenols’ calibration curve was performed by using gallic acid at concentrations of 1, 2, 3, 4, and 5 mg/L (regression coefficient R^2^ = 0.988).

The TMA content was established using the methodology described by Spinei and Oroian [17] and the data were reported as mg cyanidin-3-glucoside per gram of grape pomace extract. The TMA content was calculated using the formula reported by Spinei and Oroian [17].

The TFC was measured utilizing the method reported by Spinei and Oroian [20] and the results were represented as mg quercetin equivalents per gram of grape pomace extract. The flavonoids’ calibration curve was performed by using quercetin at concentrations of 1, 2, 3, 4, and 5 mg/L (regression coefficient R^2^ = 0.987).

The DPPH assay scavenging activity was estimated using the methodology reported by Spinei and Oroian [17]. The absorbance was determined at 515 nm against a blank. The DPPH was calculated using the formula described by Spinei and Oroian [17].

#### 2.2.5. FT-IR Analysis of Grape Pomace

FT-IR analysis of grape pomace samples was performed by the method used by Spinei and Oroian [20].

#### 2.2.6. SEM Analysis of Grape Pomace

The microstructure of the grape pomace samples was examined by scanning electron microscopy (SEM; SU-70, Hitachi, Tokyo, Japan). Dried grape pomace powder was attached to the sample spot with conductive double-sided adhesive carbon tape and investigated using an accelerating voltage of 30 kV at a magnification of 400×.

#### 2.2.7. Total Phenolic Content and Antioxidant Activity of Jelly Candy

The total phenolic content (TPC) of jelly candies was determined according to Rashad et al. [10] with slight modifications. For extraction, 2 g of jelly candies was homogenized with 20 mL of ethanol (70%, *w*/*v*) under continuous stirring at 400 rpm for 1 h. The extract was filtered in order to obtain a clear solution and TPC was determined using the Folin–Ciocalteu method described by Spinei and Oroian [17].

The DPPH assay scavenging activity of jelly candies was estimated according to Rashad et al. [10] with slight modifications. For extraction, 6 g of jelly candies was homogenized with 75 mL of ethanol (70%, *w*/*v*) under continuous stirring at 400 rpm for 1 h and then filtered. The extract was determined using the method described by Spinei and Oroian [17].

#### 2.2.8. Textural Profile of Jelly Candy

Mechanical properties (hardness (H), cohesiveness (Co), adhesiveness (Ad), springiness (S), and gumminess (G)) were determined with a Perten TVT-6700 device (Perten Instruments, Stockholm, Sweden), by using the method described by Guiné et al. [21] and Figueroa and Genovese [22].

#### 2.2.9. Sensorial Analysis of Jelly Candy

The sensory profile (e.g., color, odor, taste, elasticity, sweetness, appearance, biting, and overall acceptability) of jelly candy samples was determined using the methodology described by Rashad et al. [10]. Sensory characteristics were evaluated by 45 untrained panelists of the Faculty of Food Engineering, Stefan cel Mare University of Suceava (30 females and 15 males, aged 22 to 50 years). A 9-point hedonic scale was used for this aim; the rejection limit is less than 5 [10]. The grape pomace jellies used for the sensory analysis were prepared using silicone molds with 50 teddy bear spaces (size: 19 × 14 cm) and served at room temperature in identical beakers coded with digits.

#### 2.2.10. Microbiological Analysis of Jelly Candy

Grape pomace jellies (10 g of each sample) were homogenized with sodium chloride solution (0.85%, *w*/*v*) in order to have a final dilution of 10^−1^ for 5 min in a laboratory blender. Serial decimal dilutions were established according to the method used by Rashad et al. [10]. The total count was determined on nutrient agar at 30 °C after 48 h of incubation, while mold and yeast counts were set out on malt extract agar at 28 °C after 5 days of incubation.

### 2.3. Statistical Analysis

The results were submitted to analysis of variance (ANOVA) using XLSTAT 2023.2.1413 Statistical Software (Lumivero, Denver, CO, USA). Fisher’s least significant difference (LSD) procedure was used at the 95% confidence level. Principal component analysis (PCA) was used in order to correlate color, phytochemical, and textural characteristics (loadings) with different jelly formulations (scores) for easier viewing. The data were arranged in a matrix of 9 lines (jelly candy samples) and 10 columns (color, phytochemical, sensorial, and textural characteristics); the data were centered using variable transformation which is necessary to obtain a more representative variable and normalized by Pareto scaling (the scaling factor used is the square root of the standard deviation). The mean of the samples was used to build the PCA.

## 3. Results and Discussion

### 3.1. Influence of the Particle Size on the Proximate Composition of Grape Pomace

The structure of the grape pomace as a by-product depends on the terroir, grape variety, degree of grape ripeness, processing method, and other factors [1,9]. Therefore, all of these factors contribute to the high variability in grape pomace composition and suggest a considerable challenge for the fortification processing of the grape pomace [23]. The results of proximate analysis for grape pomace of Băbească Neagră (BN) variety (*Vitis vinifera* L.) with three different particle sizes, <125 μm, ≥125–<200 μm, and ≥200–<300 μm, are presented in Table 1; thus, the proximate composition was significantly influenced by the particle size of BN grape pomace (*p* < 0.0001).

The grape pomace with the particle size interval of ≥200–<300 µm had the highest content of insoluble dietary fiber (49.4 g/100 g oven-dried pomace powder (ODPP)), with smaller quantities of crude protein (8.4 g/100 g ODPP), crude fat (20.1 g/100 g ODPP), and moisture content (11.5 g/100 g ODPP) in comparison with grape pomace with the granularity of <125 μm (41.4 g/100 g ODPP, 7.7 g/100 g ODPP, 18.3 g/100 g ODPP, and 12 g/100 g ODPP, respectively). The obtained results are consistent with other studies, which related that the predominant constituents of the grape cell wall are lignin, cellulose, hemicellulose, and other polymeric components [1,9]. The relatively high fat content of grape pomace can be ascribed to oil from grape seeds, especially to lipophilic compounds (e.g., unsaturated fatty acids) [1,24]. The total dietary fiber (TDF) value diminished as the particle size consecutively decreased; this may be an effect of the degradation of insoluble dietary fiber (IDF), such as hemicellulose, cellulose, and lignin, in grape pomace into some tiny molecular substances [18]. Moreover, with a decrease in particle size intervals from ≥200–<300 μm to <125 μm, the IDF content decreased from 49.4 g/100 g ODPP to 41.4 g/100 g ODPP, while soluble dietary fiber (SDF) content increased from 6.9 g/100 g ODPP (<125 μm) to 7.8 g/100 g ODPP (≥125–<200 μm) and decreased to 7.6 g/100 g ODPP (≥200–<300 μm); this indicated that smaller particle size led to the redistribution of DF from the insoluble to soluble part [18]. Also, Gao et al. [18] presented the same tendency of a decrease in the SDF content of asparagus pomace with a decrease in the granularity from 109.3 to 3.18 μm. Furthermore, Antonić et al. [9] presented the proximate composition of grape pomace, obtained from studies that comprised an analysis of different grape pomace varieties, in this way: ash of 1.73–9.10 g/100 g, protein about 3.57–14.17 g/100 g, fat around 1.14–13.90 g/100 g, and TDF of 17.28–88.70 g/100 g. On the other hand, Tolve et al. [25] obtained the physicochemical composition of grape pomace (*Vitis vinifera* cv. Corvina) with a particle size of <200 μm as follows: total dietary fiber—52.3 ± 2.1 g/100 g dry matter (DM), crude protein—11.19 ± 0.97 g/100 g DM, and ash—4.17 ± 0.87 g/100 g DM, while Bender et al. [26] reported the following proximate composition of dried grape pomace (*Vitis vinifera* cv. Malbec) with a particle size of <0.59 mm: total dietary fiber—65.56 ± 0.83 g/100 g dry weight (DW), crude protein—14.17 ± 0.08 g/100 g DW, and ash—5.14 ± 0.00 g/100 g DW.

### 3.2. Influence of the Particle Size on the Functional Properties of Grape Pomace

Water-holding capacity (WHC) and swelling capacity (SC) are essential hydration characteristics of dietary fibers in food fortification and are outright attributed to the quality and functional features of food products [18]. WHC and SC belong to the insoluble polysaccharides since they can attach water by either surface tension in the pores of the matrix or ionic bonds, hydrophilic interactions, and/or hydrogen bonds [27]. As shown in Table 1, WHC varied between 4.0 (grape pomace with the particle size of <125 μm) and 7.8 g/g ODPP (grape pomace with the particle size of ≥200–<300 μm), while SC had a range from 4.1 (grape pomace with the particle size of <125 μm) to 10.1 mL/g ODPP (grape pomace with the particle size of ≥200–<300 μm); this can be explicated by the fact that the highest particle size interval of BN grape pomace contained a great amount of IDF. These results are in accordance with the data obtained by Zhang et al. [27] and Ma and Mu [28], who found that IDF could bind more water with minimal swelling. Therefore, the WHC obtained for the grape pomace with the particle size of ≥200–<300 μm (7.8 g/g) was higher than that determined for asparagus pomace (4.18–6.12 g/g), black currant pomace (2.78 g/g), cranberry pomace (3.83–3.87 g/g), lingonberry pomace (3.27–3.28 g/g), quince pomace (5.30–7.55 g/g), and sea buckthorn pomace (4.24–4.32 g/g) [18,29,30], but lower than that of grape juice pomace (8.48 g/g), *Dendrobium officinale* pomace (15.30–20.12 g/g), cranberry pomace (10.59 g/g), and deoiled red raspberry pomace (8.97 g/g) [27,31,32,33]. These data may be a result of the influence of different parameters, e.g., different particle size intervals, preparation procedure, porosity, surface characteristics, and chemical structure of the fiber [33]. Concerning the applications of by-products in the food industry, e.g., dietary fiber with a high value of WHC can be utilized as a functional ingredient in order to modify some characteristics (viscosity and texture) of composed foods, while dietary fiber with low WHC could be utilized as a sugar substitute to produce low-calorie food products (e.g., corn flakes, cookies, extruded snacks, crackers, and jelly) [27].

The water solubility index (WSI) depends on the swelling fluid, swelling power, chemical, and structure composition of the by-product [34]. The influence of particle size on the WSI is presented in Table 1; thus, the WSI was significantly influenced by the particle size of BN grape pomace (*Vitis vinifera* L.) (*p* < 0.0001). The highest value of WSI (9.4% ODPP) was obtained for the particle size interval of <125 μm, while the lowest WSI (5.2% ODPP) for the particle size of ≥200–<300 μm; in this way, the WSI of grape pomace increased significantly as its particle size decreased. Gao et al. [18] reported the same tendency for asparagus pomace samples, in which the WSI varied between 1.32 and 13.25% for 0.12 and 0.005 mm, respectively. This can be explained by the fact that grinding treatment may be capable of destroying the structure of the dietary fiber of grape pomace and releasing the soluble components, resulting in enhanced solubility [18]. Moreover, experimental characteristics, such as stirring, could influence the chemical structure of dietary fiber and the hydration process [18]. Oil-binding capacity (OBC) can be utilized in order to evaluate the functional ingredient’s property of preventing fat loss during food processing and decreasing serum cholesterol levels through adsorbing fat in the intestinal lumen [27]. As shown in Table 1, the OBC of the grape pomace varied between 1.9 (grape pomace with the particle size of ≥200–<300 μm) and 2.7 g/g ODPP (grape pomace with the particle size of <125 μm); these data can be related to the presence of lignin (insoluble dietary fiber) which plays a role in oil absorption [18,27]. Certainly, the OBC of the BN grape pomace obtained in this study was relatively higher in comparison with dietary fiber extracted from grape juice pomace (0.65 g/g), asparagus pomace with a particle size of 0.03 mm (2.45 g/g), black currant pomace (1.14 g/g), cranberry pomace (1.57 g/g), lingonberry pomace (1.46 g/g), grape pomace (1.42 g/g), and sea buckthorn pomace (1.09 g/g) [18,26,27,29]. Previous studies reported a higher value of OHC for citrus peel dietary fiber, such as orange dietary fiber—3.62 g/g and grapefruit dietary fiber—8.20 g/g [35]. This resulted most probably as a consequence of fiber composition, surface properties, particle size, hydrophobic nature of particles, and overall charge density [27]. Therefore, the functional properties of grape pomace, e.g., WHC and SC, presented a typical exponential curve with a maximal value for upper-size particles, while OBC showed this curve for lower-size particles.

### 3.3. Influence of the Particle Size on the Color of Grape Pomace

**Figure 1:**
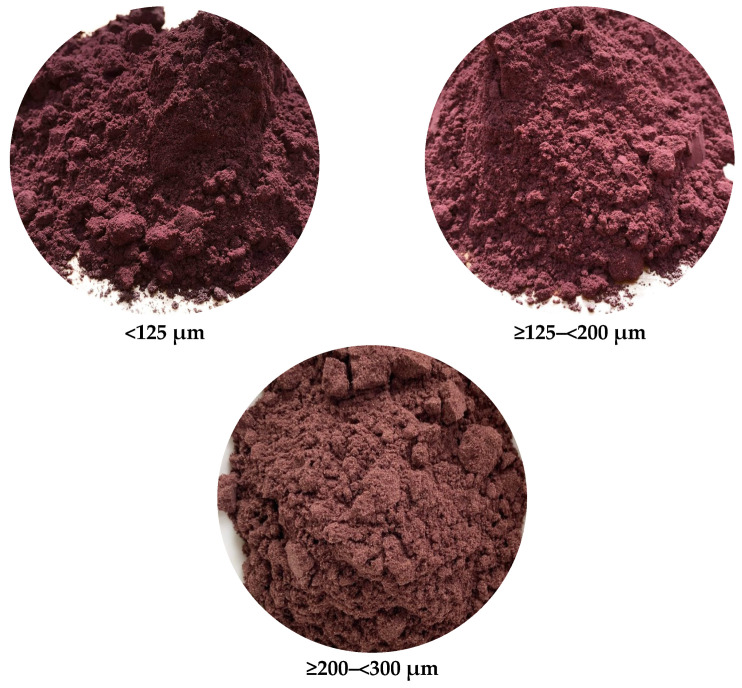
Images of Băbească Neagră grape pomace under the influence of different granularities.

The color of grape pomace, particularly red varieties, is determined by the amount of anthocyanins, localized mostly in the skin [4]. Furthermore, color is affected by different physicochemical parameters, e.g., pH, temperature, particle size of material, and time [4]. The influence of particle size on the color parameters (L*, C*_ab_, and h*_ab_) of grape pomace is presented in Table 1; thus, the L*, C*_ab_, and h*_ab_ were not significantly influenced by the granularity of grape pomace (*p* > 0.05). The highest value of L* (29.9) and C*_ab_ (14.9) was obtained for grape pomace with the granularity of ≥200–<300 µm, while the lowest L* (17.6) and C*_ab_ (11.2) by <125 µm grape pomace. Therefore, grape pomace samples showed a color ranging from blue-purple to purple in conformity with the CIE chromaticity diagram. This can be explained by the fact that anthocyanins interact with other phenolics to create co-pigments which improve color stability during and after the winemaking process [36]. Moreover, grape pomace is rich in flavonoids, polyphenols, and anthocyanins with a high antioxidant capacity and distinct phenolic compounds [1]. In order to concur with the results presented for color parameters (L*, C*_ab_, and h*_ab_), images of grape pomace samples with different particle sizes are shown in Figure 1.

### 3.4. Phytochemical Characterization of Grape Pomace Extract

Vinification by-products consist of a relatively high content of polyphenolic substances, which depends on the grape variety (white or red), processing conditions (e.g., type of maceration), and the part of vegetal material (seeds, skins, etc.) [37,38]. The phytochemical characterization of grape pomace (Băbească Neagră variety) extract was performed by evaluating the following parameters: TPC, TMA content, TFC, and antioxidant activity (Table 1). The highest value of TPC, TMA content, and DPPH was obtained for the granularity of <125 μm (146 mg gallic acid equivalent (GAE)/g ODPP, 11.4 mg cyanidin-3-glucoside (C3G)/g ODPP, and 67.10% ODPP, respectively), while the lowest for the particle size of ≥200–<300 µm (135 mg GAE/g ODPP, 6.9 mg C3G/g ODPP, and 48.6% ODPP, respectively). This can be explained by the fact that particle size intervals significantly influence the extraction of bioactive compounds (*p* < 0.0001). Moreover, the phytochemical characterization (TPC, TMA content, and DPPH) is affected by several factors, such as drying, extraction process, solvent, and extraction temperature [39]. The same results were obtained for Cabernet Franc pomace (153.8 mg GAE/g for TPC and 91.7 mg catechin equivalents (CE)/g for TFC) [40]. On the other hand, the obtained results for grape pomace extract are higher than the TPC reported by Carmona-Jiménez et al. [41] for different grape pomace varieties, such as Tempranillo (49.70 mg GAE/g), Cabernet Sauvignon (44.73 mg GAE/g), Tintilla de Rota (43.85 mg GAE/g), Syrah (44.11 mg GAE/g), and Petit Verdot (46.57 mg GAE/g). Also, Barriga-Sánchez et al. [39] reported a lower TPC (54.36 mg GAE/g) for Black Borgoña (*Vitis labrusca* L.) pomace using ethanol–water as solvent.

### 3.5. FT-IR Spectra of Grape Pomace

Different organic functional groups, notably N–H, O–H, and C=O, are determined by FT-IR spectroscopy. Consequently, FT-IR spectra were obtained to study the influence of particle size on the chemical structure of Băbească Neagră (BN) grape pomace (*Vitis vinifera* L.). Common absorption bands of phenolic compounds and polysaccharides that are found in the structure of grape pomace are shown in Figure S1 of the Supplementary Materials. The grape pomace samples had stretching bands of O–H and O(6)H···O(3) at 3315–3310 cm^−1^ which were attributed to vibrations of the functional group (–OH) of the phenolic and polysaccharide structures, specifically insoluble dietary fibers, e.g., cellulose and hemicellulose [26]. Furthermore, the C–H stretching vibration peak at 2918 cm^−1^ was assigned to a methylene group of polysaccharides [26]. The band position at 1741 cm^−1^ corresponded to the stretching band of carbonyl group C=O, which indicated the presence of the galloyl group or uronic acid [26,42]. Stretching bands at 1625–1605 cm^−1^ showed the presence of benzene rings in lignin (C–H bonds) [26]. The band positions of 1510 cm^−1^, 1435 cm^−1^, and 1377 cm^−1^ corresponded to C=C stretching vibration, antisymmetric, and symmetric in-plane bending of –CH_3_, respectively [43]. The peaks at 1132 cm^−1^ and 1064 cm^−1^ were related to aromatic C–O–C stretching vibrations (presence of cellulose and hemicellulose) and C–O stretching bands (presence of pyranose ring), respectively [26,44]. Moreover, these band positions are attributed to polysaccharide structures [40]. The absorptive peaks at 905 cm^−1^, 842 cm^−1^, and 789 cm^−1^ were ascribed to β-*D*-pyranose, the phenolic compounds, and the aromatic ring breathing manner, respectively [42]. The spectral profile of BN grape pomace with different particle size intervals indicated that the position and number of the characteristic absorption peaks of samples did not change significantly.

### 3.6. Microstructural Analysis by SEM

**Figure 2:**
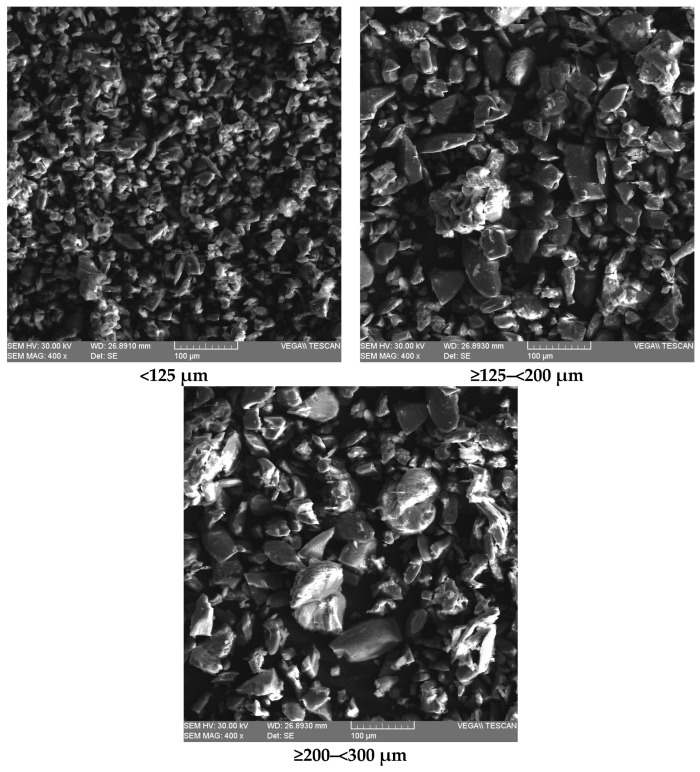
SEM images of Băbească Neagră grape pomace under the influence of different granularities.

SEM is an efficacious technique for analysis of the crystallography, shape, size, composition, and other physicochemical properties of different materials [45]. The morphology of Băbească Neagră (BN) grape pomace was investigated using SEM. As shown in Figure 2, a couple of changes were examined among the grape pomace samples with different particle sizes; the grape pomace with the particle size of <125 μm had a small fibrous, compact, and reticular structure, while other samples presented various rough and irregular surfaces in combination with fibrous structures. According to Pala et al. [46] and Gowman et al. [47], the irregular surface of the samples is attributed to the strong internal bonds in the structure of the lignocellulosic fibers. Moreover, it can be explained by the fact that the grape pomace consisted of seeds, skins, and stems which have different components. The grape pomace skins are flatter, but become irregular after the drying process, while the stems are more fibrous because of the amount of insoluble dietary fibers. The grape pomace seeds are irregular and, like stems, have fibrous and porous surfaces [47]. Therefore, different particle size intervals influenced the surface morphology of grape pomace samples and resulted in different fibrous structures, as presented in the SEM images. The porous surface gives additional space for the formation of hydrogen bonding by water molecule content; this feature could increase the OBC and WHC of the grape pomace samples [48]. The same results were reported by Drevelegka and Goula [49], who established that grape pomace had a solid and relatively organized cell structure. Also, Baldán et al. [48] explained that different particle sizes were related to the hydrophilic components of the grape pomace, e.g., fiber, polysaccharides, protein, and pigments.

### 3.7. Phytochemical Parameters and Proximate Composition of Grape Pomace Aqueous Extract

The proximate composition and phytochemical parameters (DPPH assay scavenging activity and TPC) of grape pomace aqueous extract are shown in Table 2. The parameters of proximate composition, such as moisture, ash, carbohydrates, and total dietary fiber (TDF), were significantly influenced by the granularity of grape pomace extract (*p* < 0.0001).

The highest values for moisture content (89.3% ODPP) and ash (0.12 g/100 mL ODPP) were obtained for the grape pomace extract with the granularity of ≥200–<300 µm, while the lowest values were obtained for the grape pomace extract with the granularity of <125 µm (84.1% ODPP and 0.02 g/100 mL ODPP for moisture content and ash, respectively). Crude fat was not found in any sample; this can be explained by the fact that fats did not pass into the water extract and did not have any polar bonds. Moreover, only the grape pomace extract with the granularity of ≥200–<300 µm had crude protein (0.23 g/100 mL ODPP) in its composition. The presence of protein can be attributed to the constituents of grape pomace, especially due to inflorescence architectures and grape seeds which contain an amount of 6.1% and 11% protein, respectively [1]. The highest contents of carbohydrates (15.9 g/100 mL ODPP) and TDF (12.4 g/100 mL ODPP) were obtained for the grape pomace extract with the granularity of <125 µm, while the lowest for the grape pomace extract with the granularity of ≥200–<300 µm (10.3 g/100 mL ODPP and 9.7 g/100 mL ODPP, respectively). Therefore, viable sources of carbohydrates that are presented in grape pomace extract are water-soluble monosaccharides (e.g., fructose and glucose) [1,4].

The grape pomace extracts showed a great quantity of TPC (a range between 107 and 126 mg GAE/mL ODPP) as a result of hydrophilic compounds (e.g., phenolic acids) which are abundant in the solid parts of grape pomace, especially in grape skins and grape seeds [2,4]. The DPPH assay scavenging activity showed a similar tendency to TPC; these results are in accordance with other studies [24,26].

### 3.8. Physicochemical Parameters of Jelly Candy

The physicochemical parameters, such as color parameters (L*, C*_ab_, and h*_ab_), DPPH assay scavenging activity, and TPC were determined for jelly candies (Table 3); thus, the L*, C*_ab_, and h*_ab_ were significantly influenced by the granularity of grape pomace (*p* < 0.0001).

**Figure 3:**
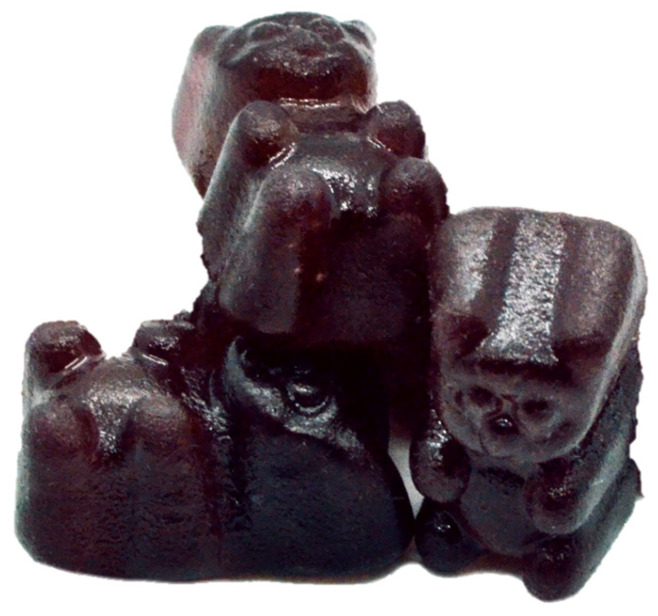
Jelly samples obtained from grape pomace extract, gelatin, and stevia.

The highest values for L* (42.7), C*_ab_ (18.1), and h*_ab_ (48.9) were obtained for jelly formulation F3–10 (grape pomace extract with the granularity of ≥200–<300 μm and 10 g of gelatin), while the lowest for F1–7 (grape pomace extract with the granularity of <125 μm and 7 g of gelatin). Thus, jelly samples showed a color ranging from red-purple to purple in conformity with the CIE chromaticity diagram. The color of the jelly candies obtained from the grape pomace extract with the particle size of <125 μm and 7 g of gelatin got darker after the mixing step; this may be explained by the quick dissolution of grape pomace with the diameter of <125 μm, and also, by the presence of polyphenols and other water-soluble compounds (e.g., pigments). The resulting color is also directly correlated to the chemical structures of anthocyanins present in grape pomace extract, the presence of different co-pigmentations, and the chemical reactions implicating these components during the homogenization of the mixture, as well as the concentration of gelling agent (gelatin) and temperature used in that process [50,51]. In order to concur with the results presented for the color parameters (L*, C*_ab_, and h*_ab_), images of jelly samples are presented in Figure 3.

TPC and DDPH assay scavenging activity of jelly candies are greatly dependent on the gelling agent, solvent nature (water and ethanol), extraction method, and formulation procedure [39]. The highest value of TPC was found for jelly samples obtained from the grape pomace extract with the particle size of <125 μm and with the addition of 7 g of gelatin. DPPH values varied from 29.9 to 65.8% for F3–10 and F1–7, respectively. Statistically, DPPH antioxidant activity values showed a similar tendency to TPC. It is well known that polyphenol content is definitely correlated with antioxidant capacity, particularly in the case of fruit, fruit juices, and extracts from different pomaces [50]. Mainly, it can be described that the higher the content of the gelling agent, the lower the functional properties and bioactive compounds (e.g., polyphenols) of jellies [50,52]. On the other hand, the addition of stevia in the jelly formulations increases the antioxidant capacity of the samples because stevia contains higher amounts of antioxidant compounds (hydroxyl radicals, free radicals, and superoxide anion radical scavenging activities) [53]. Similar results for DPPH and TPC were reported by Cano-Lamadrid et al. [50] for jelly candies based on pomegranate juice “Mollar de Elche”.

### 3.9. Textural Parameters of Jelly Candy

Textural analysis was performed using the methodology reported by Garrido et al. [54]. The values obtained for textural parameters, such as hardness, cohesiveness, adhesiveness, springiness, and gumminess are presented in Table 3. Hardness relates to the force necessary to obtain a given deformation [18]. The data obtained for hardness were statistically different among samples (*p* < 0.0001), with the highest value (17.1 N) for F2–10 (grape pomace extract with the particle size of ≥125–<200 μm and 10 g of gelatin) and the lowest (6.1 N) for F3–7 (grape pomace extract with the particle size of ≥200–<300 μm and 7 g of gelatin); these data can be related to the concentration of the gelling agent (gelatin) and the composition of grape pomace extracts with different particle sizes (e.g., protein, carbohydrates, and fiber content). Previous studies reported a higher value of hardness for strawberry and mint jelly gum (26.7–30.3 N), strawberry and anise jelly gum (24.5–26.7 N), raspberry and mint jelly gum (22.8–27.7 N), blueberry and mint (25.1–29.3 N), grape seed soft candies (11.34–47.53 N), and grape skin soft candies (16.79–24.08 N) [15,18]. On the other hand, Garrido et al. [54] obtained a lower value of hardness of apple jelly (0.6–5.2 N). Additionally, the interaction of proteins and carbohydrates which are presented in grape pomace extract has a significant influence on enhancing hardness and other textural parameters, such as springiness and cohesiveness [22].

Cohesiveness refers to the internal bonds of the food that keep the mass cohesive and prevent it from disintegrating [21]. The values of cohesiveness ranged from 0.23 for F2–7 (grape pomace extract with the particle size of ≥125–<200 μm and 7 g of gelatin) to 0.25 for F3–10 (grape pomace extract with the particle size of ≥200–<300 μm and 10 g of gelatin). The fiber content (9.7–12.4 g/100 mL ODPP) of aqueous extract had a positive effect on cohesiveness; fibers cause a significant increase in cohesiveness [22]. Similarly, Akesowan [55] reported a range between 0.202 and 0.322 for the cohesiveness of konjac jelly, while Garrido et al. [54] obtained higher values (0.37–0.53) for apple jelly; this can be explained by the fact that the more gelling agent existed in the gel system, the more cohesive the jelly candy became [55].

Adhesiveness refers to the force needed to eliminate the material that adheres to a surface [21]. The results obtained for adhesiveness were statistically different among samples (*p* < 0.01), with the highest value (−3.3 N × s) for F3–10 (grape pomace extract with the particle size of ≥200–<300 μm and 10 g of gelatin) and the lowest (−3.0 N × s) for F1–7 (grape pomace extract with the particle size of <125 μm and 7 g of gelatin). Several factors, e.g., the amount of sugar naturally present in grape pomace, pectin content, acidity, and concentration of gelling agent may explain the variation in adhesiveness among samples prepared with different particle sizes of grape pomace. On the other hand, Rios de Souza et al. [56] reported higher values ((−12.75)–(−37.50) N × s) for blackberry jellies from different cultivars (Choctaw, Brazos, Cherokee, Comanche, Caingangue, Guarani, and Tupy). In addition, other parameters, such as phytochemical (e.g., TPC) and proximate composition (e.g., moisture content, TDF, and crude protein) of the grape extract can affect the textural characteristics by influencing the yield, cooking time, and water activity of the final jelly candies [56].

Springiness is related to the ability of shape recovery after compression and represents the rate at which a deformed material returns to the initial form after the force is removed [54]. The values of springiness ranged from 0.83 for F1–10 (grape pomace extract with the particle size of <125 μm and 10 g of gelatin) to 0.89 for F3–10 (grape pomace extract with the particle size of ≥200–<300 μm and 10 g of gelatin). Similarly, Rios de Souza et al. [56] reported a range between 0.93 and 0.98 for the springiness of blackberry jellies from different cultivars (Brazos, Caingangue, Cherokee, Choctaw, Comanche, Guarani, and Tupy), while Altınok et al. [15] obtained a lower value (0.484) for grape skin soft candies with a particle size of 100 μm. The results showed that springiness was most reproducible in relation to the other textural responses, e.g., cohesiveness and hardness. Moreover, the highest particle size interval (≥200–<300 μm) influenced the springiness due to the fiber content of grape pomace and gelling agent, which subsequently affects the melting properties [22,55].

Gumminess has been interpreted as the energy involved in disintegrating a semi-solid food product into a state ready for swallowing [50]. The highest value (55.2 N) for F3–8.5 (grape pomace extract with the particle size of ≥200–<300 μm and 8.5 g of gelatin) and the lowest (48.9 N) for F1–7 (grape pomace extract with the particle size of <125 μm and 7 g of gelatin); these data can be related to the gelatin dose, treatment, and the presence of sugars [21]. The increase in the gelatine dose enhanced gumminess, cohesiveness, and hardness; these results are in accordance with the data obtained by Mutlu et al. [57]. Moreover, the results of the texture profile analysis showed that the particle size of plant material (i.e., grape pomace) is a significant factor in the formulation of jelly candy.

### 3.10. Sensorial Analysis of Jelly Candy

**Figure 4:**
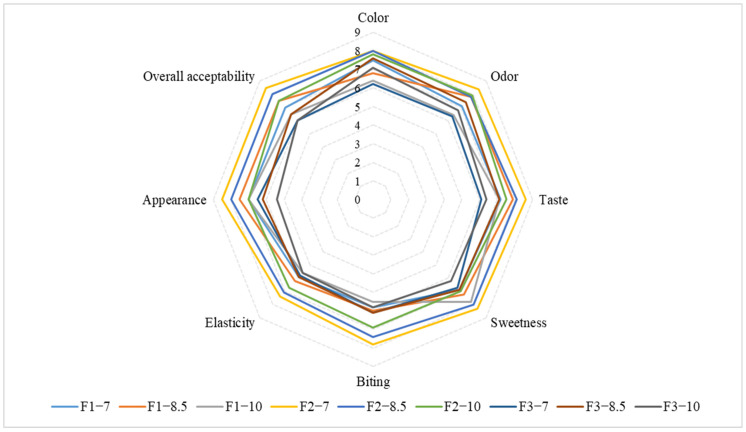
Radar chart of sensory analysis of jelly samples (F1—grape pomace extract with the granularity of <125 μm; F2—grape pomace extract with the granularity of ≥125–<200 μm; F3—grape pomace extract with the granularity of ≥200–<300 μm; 7—7 g of gelatin; 8.5—8.5 g of gelatin; 10—10 g of gelatin).

The radar chart of sensory analysis (e.g., color, odor, taste, elasticity, sweetness, appearance, biting, and overall acceptability) of the grape pomace jellies is presented in Figure 4; the quality sensory characteristics of jelly samples were expressed as appealing. Generally, the jelly candies presented good sensory acceptance for all characteristics, with average scores ranging between the hedonic terms “I liked it slightly” and “I liked it very much”. The textural parameters (biting and elasticity) were presented as easy to bite and soft by the majority of the panelists. The jelly formulations F3–7 and F3–10 recorded the lowest value of overall acceptability due to the presence of the highest contents of insoluble fiber and fats, which have an unpleasant texture and taste. The highest values for taste, sweetness, odor, and overall acceptability were obtained for jelly formulations F2–7, F2–8.5, and F1–8.5; these results can be attributed to the lower sizes of grape pomace particles which are correlated with a lower content of fats and insoluble fiber. The results showed that grape pomace jellies have a gummy and smooth texture with a gelatinous appearance and sweet taste, which indicates that the formulation and ratios chosen for the gelling agent (gelatin) were adequate and presented a positive influence on the sensory characteristics of the final product. The same tendency of sensorial parameters was reported for jelly candies based on pomegranate juice “Mollar de Elche”, strawberry and red beetroot jelly candies, and strawberry jellies from different cultivars (Camino Real, Festival, Camarosa, Oso Grande, Albion, and San Andreas) [10,50,58]. These findings can be highlighted for production by focusing attention on the fact that grape pomace jelly is a product based on stevia (zero-calorie sweetener), does not contain added sugar (only natural sweeteners, i.e., water-soluble monosaccharides present in grape pomace extract), and artificial dyes.

### 3.11. Microbiological Analysis

The results of the microbiological analysis (total plate count, mold, and yeast counts) of grape pomace jellies are presented in Table 3. The total plate count (TP count) and mold and yeast counts (MY counts) were significantly influenced by the particle size of BN grape pomace and the ratio of gelatin used for jelly formulation (*p* < 0.0001). The highest values for TP count (95.3 CFU/mL) and MY counts (74.1 CFU/mL) were obtained for jelly formulation F3–10; this can be explained by the fact that F3–10 had the highest contents of fats and proteins. Moreover, gelatin used as the gelling agent may facilitate the formation of mold and yeast due to gelatin consisting of 98–99% protein, which can be a source of microorganisms [59]. According to Thompson [59], jellies have values for water activity ranging between 0.65 and 0.75, which means that jellies are susceptible to spoilage by xerophilic molds and osmophilic yeasts. At the same time, there was no growth in other jelly samples, which may be a result of the high phenolic content of grape pomace. On the other hand, Ismawati et al. [60] reported a higher TP count (2.1 × 10^2^ CFU/mL) and MY counts (8.9 × 10^1^ CFU/mL) for seaweed jelly candy.

### 3.12. Principal Component Analysis

**Figure 5:**
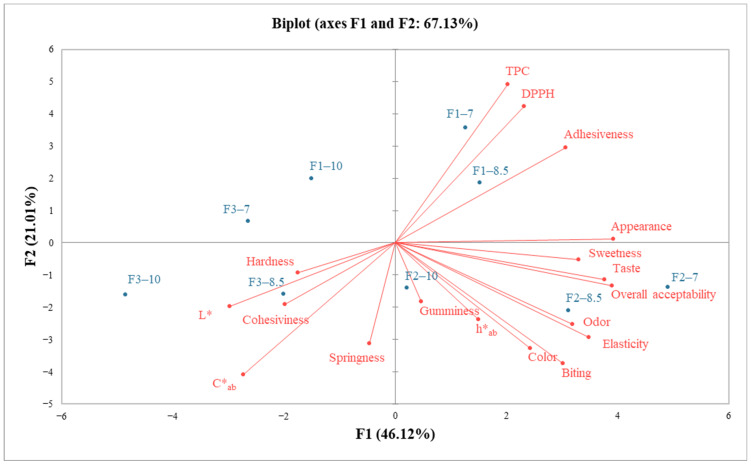
PCA biplot showing the correlation between scores (jelly samples, ●) and loadings (color parameters, phytochemical characteristics, sensorial, and textural parameters, ●); F1—grape pomace extract with the granularity of <125 μm; F2—grape pomace extract with the granularity of ≥125–<200 μm; F3—grape pomace extract with the granularity of ≥200–<300 μm; 7—7 g of gelatin; 8.5—8.5 g of gelatin; 10—10 g of gelatin; L*—luminosity; h*_ab_—hue; C*_ab_—chroma; TPC—total phenolic content.

Principal component analysis (PCA) of the experimental findings was utilized to decompose the dataset into scores (jelly candies) and loadings (color parameters, phytochemical characteristics, and sensorial and textural parameters). As presented in Figure 5, the principal components described the better part of variability, with a total variance contribution of 67.13% (F1—1: 46.12%, F2—2: 21.01%). The biplot presents that the most of obtained characteristics demonstrated a significant influence on the distinctions among jelly samples. The jelly samples F2–7, F2–8.5, and F2–10 were correlated with sensorial parameters (e.g., sweetness, taste, odor, elasticity, color, and overall acceptability), gumminess, and h*_ab_, while formulations F3–8.5 and F3–10 were associated with hardness, cohesiveness, L*, and C*_ab_. The arrangement of the F1–7 and F2–8.5 samples more to the right was related to their high values of adhesiveness, and phytochemical characteristics (TPC and DPPH), while F3–7 and F1–10 jelly candies were not correlated with any of the studied parameters.

## 4. Conclusions

In recent times, grape pomace has been used as a fortifying agent for a lot of food products (plant foodstuffs, dairy products, meat, and fish products). Concerning the valorization of by-products, it is very important to improve the nutritional value of products and obtain new products. Therefore, Băbească Neagră grape pomace was used as a fortifying agent in jelly candy. For a better understanding of how to fortify grape pomace, the proximate composition, functional properties, and physicochemical characteristics were analyzed. The obtained results were in agreement with FT-IR and SEM analysis of grape pomace. Furthermore, the grape pomace extract was analyzed in terms of phytochemical parameters and proximate composition; the results contributed to a more complete explanation of the jelly formulation and characterization of the final product. So, by-product-based jelly candies were formulated and had the following composition: grape pomace extract with different particle sizes (<125 μm, ≥125–<200 μm, and ≥200–<300 μm), gelatin (7, 8.5, and 10 g), and stevia. The obtained results showed that grape pomace jellies with the particle size of ≥125–<200 μm and different concentrations of gelatin had the greatest acceptance in terms of sensorial properties (e.g., sweetness, taste, odor, elasticity, color, and overall acceptability), while the highest value of phytochemical characteristics (156 mg GAE/g for total phenolic content and 65.8% inhibition of antioxidant activity) was acquired for jelly candy formulated with grape pomace extract (<125 μm) and 7 g of gelatin. The application of grape pomace extract as a natural ingredient in the formulation of jelly candy resulted in an improvement with a great concentration of polyphenols, along with uniform purple color, pleasant taste, and a high acceptance in terms of textural parameters (i.e., hardness, cohesiveness, adhesiveness, springiness, and gumminess). Thereby, all the data indicate the potential of Băbească Neagră grape pomace for use as a fortifying agent in jelly candies.

## Figures and Tables

**Table 1:** Proximate composition, physicochemical, and functional properties of grape pomace.

Parameter	Particle Size (µm)			*p*-Value
	<125	≥125–<200	≥200–<300	
Proximate composition				
Moisture (g/100 g ODPP)	12.0 (0.34) ^a^	11.9 (0.26) ^b^	11.5 (0.29) ^c^	*p* < 0.0001
Crude protein (g/100 g ODPP)	7.7 (0.28) ^c^	8.0 (0.33) ^b^	8.4 (0.42) ^a^	*p* < 0.0001
Crude fat (g/100 g ODPP)	18.3 (0.22) ^c^	19.4 (0.25) ^b^	20.1 (0.18) ^a^	*p* < 0.0001
Ash (g/100 g ODPP)	1.9 (0.14) ^a^	1.8 (0.19) ^b^	1.6 (0.27) ^c^	*p* < 0.0001
Carbohydrates ^A^ (g/100 g ODPP)	60.0 (0.27) ^a^	58.8 (0.21) ^b^	58.2 (0.34) ^c^	*p* < 0.0001
TDF (g/100 g ODPP)	48.2 (0.36) ^c^	55.0 (0.32) ^b^	57.0 (0.26) ^a^	*p* < 0.0001
IDF (g/100 g ODPP)	41.4 (0.19) ^c^	47.1 (0.23) ^b^	49.4 (0.28) ^a^	*p* < 0.0001
SDF (g/100 g ODPP)	6.9 (0.23) ^b^	7.8 (0.15) ^a^	7.6 (0.19) ^a^	*p* < 0.0001
Functional properties				
WHC (g/g ODPP)	4.0 (0.13) ^c^	5.1 (0.17) ^b^	7.8 (0.19) ^a^	*p* < 0.0001
OBC (g/g ODPP)	2.7 (0.08) ^a^	2.1 (0.12) ^b^	1.9 (0.07) ^c^	*p* < 0.0001
SC (mL/g ODPP)	4.1 (0.28) ^c^	6.9 (0.32) ^b^	10.1 (0.37) ^a^	*p* < 0.0001
WSI (% ODPP)	9.4 (0.11) ^a^	8.3 (0.18) ^b^	5.2 (0.09) ^c^	*p* < 0.0001
Color parameters				
L*	17.6 (0.14) ^b^	25.1 (0.11) ^a^	29.9 (0.09) ^a^	*p* > 0.05
h*_ab_	39.9 (0.21) ^a^	38.1 (0.28) ^b^	38.7 (0.34) ^ab^	*p* > 0.05
C*_ab_	11.2 (0.17) ^b^	14.3 (0.12) ^ab^	14.9 (0.07) ^a^	*p* > 0.05
Phytochemical characteristics				
TPC (mg GAE/g ODPP)	146 (0.22) ^a^	136 (0.32) ^b^	135 (0.37) ^b^	*p* < 0.0001
TMA content (mg C3G/g ODPP)	11.4 (0.46) ^a^	9.2 (0.41) ^b^	6.9 (0.38) ^c^	*p* < 0.0001
TFC (mg QE/g ODPP)	19.0 (0.13) ^b^	21.4 (0.26) ^a^	17.5 (0.17) ^c^	*p* < 0.0001
DPPH (% inhibition ODPP)	67.1 (0.19) ^a^	50.4 (0.54) ^b^	48.6 (0.68) ^c^	*p* < 0.0001

Mean values (*n* = 9) and standard deviation are shown in brackets. ^a–c^—different letters in the same row indicate significant differences among samples (*p* < 0.0001) in conformity with the Fisher test with α = 0.05. ^A^—carbohydrate content was determined by the difference method; TDF—total dietary fiber; IDF—insoluble dietary fiber; SDF—soluble dietary fiber; WHC—water-holding capacity; OBC—oil-binding capacity; SC—swelling capacity; WSI—water solubility index; L*—luminosity; h*_ab_—hue; C*_ab_—chroma; TPC—total phenolic content; TMA—total monomeric anthocyanin content; TFC—total flavonoid content; GAE—gallic acid equivalent; C3G—cyanidin-3-glucoside; QE—quercetin equivalent; ODPP—oven-dried pomace powder.

**Table 2:** Proximate composition and phytochemical parameters of grape pomace aqueous extract.

Parameter	Particle Size (µm)			*p*-Value
	<125	≥125–<200	≥200–<300	
Proximate composition				
Moisture (% ODPP)	84.1 (0.13) ^c^	86.7 (0.16) ^b^	89.3 (0.14) ^a^	*p* < 0.0001
Crude protein (g/100 mL ODPP) *	n.d.	n.d.	0.23 (0.01) ^a^	*p* < 0.0001
Crude fat (g/100 mL ODPP) *	n.d.	n.d.	n.d.	–
Ash (g/100 mL ODPP) *	0.02 (0.02) ^c^	0.05 (0.02) ^b^	0.12 (0.01) ^a^	*p* < 0.0001
Carbohydrates ^A^ (g/100 mL ODPP) *	15.9 (0.06) ^a^	13.2 (0.08) ^b^	10.3 (0.11) ^c^	*p* < 0.0001
TDF (g/100 mL ODPP) *	12.4 (0.11) ^c^	10.6 (0.12) ^b^	9.7 (0.16) ^a^	*p* < 0.0001
Phytochemical characteristics				
TPC (mg GAE/mL ODPP)	126 (0.22) ^a^	107 (0.32) ^c^	109 (0.37) ^b^	*p* < 0.0001
DPPH (% inhibition ODPP)	60.8 (0.17) ^a^	49.6 (0.25) ^b^	42.5 (0.32) ^c^	*p* < 0.0001

Mean values (*n* = 9) and standard deviation are shown in brackets. ^a–c^—different letters in the same row indicate significant differences among samples (*p* < 0.0001) in conformity with the Fisher test with α = 0.05. ^A^—carbohydrate content was determined by the difference method; TDF—total dietary fiber; TPC—total phenolic content; GAE—gallic acid equivalent; ODPP—oven-dried pomace powder; *—The data was expressed as g per 100 mL of extract produced for each of the studied fractions from ODPP; n.d.—not detected.

**Table 3:** Physicochemical and textural properties of jelly candy.

Parameter	Samples									*p*-Value
	F1–7	F1–8.5	F1–10	F2–7	F2–8.5	F2–10	F3–7	F3–8.5	F3–10	
Color parameters										
L*	29.9 (0.13) ^d^	32.5 (0.17) ^c^	39.4 (0.16) ^ab^	29.2 (0.14) ^d^	33.4 (0.19) ^c^	40.2 (0.19) ^ab^	33.1 (0.12) ^c^	36.2 (0.08) ^b^	42.7 (0.11) ^a^	*p* < 0.0001
h*_ab_	40.7 (0.14) ^d^	42.1 (0.17) ^cd^	43.6 (0.17) ^c^	41.3 (0.13) ^cd^	44.7 (0.14) ^c^	46.3 (0.15) ^bc^	45.6 (0.18) ^bc^	47.1 (0.13) ^b^	48.9 (0.15) ^a^	*p* < 0.0001
C*_ab_	15.1 (0.30) ^c^	16.2 (0.22) ^bc^	16.8 (0.23) ^bc^	15.9 (0.17) ^c^	16.9 (0.21) ^bc^	17.2 (0.16) ^b^	16.8 (0.24) ^bc^	17.5 (0.29) ^b^	18.1 (0.27) ^a^	*p* < 0.0001
Phytochemical characteristics										
TPC (mg GAE/g ODPP)	156 (1.42) ^a^	148 (1.29) ^b^	138 (2.01) ^c^	130 (2.13) ^d^	129 (1.08) ^d^	127 (1.37) ^d^	120 (1.23) ^e^	118 (0.99) ^e^	115 (1.14) ^e^	*p* < 0.0001
DPPH (% inhibition ODPP)	65.8 (0.98) ^a^	63.3 (1.10) ^ab^	58.3 (2.18) ^b^	48.1 (1.15) ^c^	46.4 (0.57) ^c^	46.4 (0.69) ^c^	30.1 (1.77) ^d^	30.0 (2.13) ^d^	29.9 (2.27) ^d^	*p* < 0.0001
Textural parameters										
H (N)	6.3 (0.13) ^c^	11.1 (2.13) ^b^	16.7 (1.10) ^a^	6.5 (0.27) ^c^	10.8 (1.08) ^b^	17.1 (0.98) ^a^	6.1 (0.24) ^c^	10.7 (1.37) ^b^	16.5 (0.46) ^a^	*p* < 0.0001
Co (adim.)	0.23 (0.02) ^a^	0.24 (0.02) ^a^	0.24 (0.01) ^a^	0.23 (0.06) ^a^	0.24 (0.06) ^a^	0.25 (0.05) ^a^	0.23 (0.01) ^a^	0.24 (0.03) ^a^	0.25 (0.07) ^a^	*p* > 0.05
Ad (N × s)	−2.9 (0.08) ^a^	−2.9 (0.10) ^a^	−3.0 (0.06) ^a^	−3.0 (0.13) ^a^	−3.0 (0.08) ^a^	−3.1 (0.07) ^a^	−3.2 (0.07) ^ab^	−3.2 (0.11) ^ab^	−3.3 (0.06) ^b^	*p* < 0.01
S (adim.)	0.85 (0.03) ^b^	0.84 (0.01) ^b^	0.83 (0.01) ^b^	0.88 (0.01) ^a^	0.85 (0.01) ^b^	0.84 (0.02) ^b^	0.85 (0.03) ^b^	0.86 (0.02) ^b^	0.89 (0.01) ^a^	*p* < 0.01
G (N)	48.9 (0.12) ^d^	54.2 (0.18) ^b^	54.0 (0.17) ^b^	53.1 (0.15) ^c^	54.2 (0.12) ^b^	54.4 (0.16) ^b^	55.1 (0.18) ^a^	55.2 (0.12) ^a^	49.6 (0.11) ^d^	*p* < 0.001
Microbiological tests										
TP count (CFU/mL)	n.d.	n.d.	n.d.	n.d.	n.d.	n.d.	69.3 (0.14) ^c^	74.6 (0.17) ^b^	95.3 (0.23) ^a^	*p* < 0.0001
MY counts (CFU/mL)	n.d.	n.d.	n.d.	n.d.	n.d.	n.d.	51.6 (0.13) ^c^	53.3 (0.18) ^b^	74.1 (0.21) ^a^	*p* < 0.0001

Mean values (*n* = 9) and standard deviation are shown in brackets. ^a–e^—different letters in the same row indicate significant differences among samples (*p* < 0.0001) in conformity with the Fisher test with α = 0.05. F1—grape pomace extract with the granularity of <125 μm; F2—grape pomace extract with the granularity of ≥125–<200 μm; F3—grape pomace extract with the granularity of ≥200–<300 μm; 7—7 g of gelatin; 8.5—8.5 g of gelatin; 10—10 g of gelatin; L*—luminosity; h*_ab_—hue; C*_ab_—chroma; TPC—total phenolic content; GAE—gallic acid equivalent; H—hardness; Co—cohesiveness; Ad—adhesiveness; S—springiness; G—gumminess; TP Count—total plate count; MY Counts—mold and yeast counts; ODPP—oven-dried pomace powder; n.d.—not detected.

## Data Availability

Data is contained within the article or supplementary material.

## Supplementary Materials

The following supporting information can be downloaded at: https://www.mdpi.com/article/10.3390/foods13010098/s1, Table S1. The list and amount of ingredients used for jelly formulation; Figure S1. FT-IR spectra of Băbească Neagră grape pomace under the influence of different granularity.
