# Supplementary material for: Characterization of Băbească Neagră Grape Pomace and Incorporation into Jelly Candy: Evaluation of Phytochemical, Sensory, and Textural Properties
Source: Foods. 2023 Dec 27;13(1):98. doi: 10.3390/foods13010098 (PMC10778775; doi:10.3390/foods13010098)
Supplement: Supplementary file 1 [file foods-13-00098-s001.zip › foods-2719143-Supplementary.pdf]

Supplementary Materials

Table S1. The list and amount of ingredients used for jelly formulation.

Ingredients	Samples								
	<125 µm			≥125 – <200 µm			≥200 – <300 µm		
	F1-7	F1-8.5	F1-10	F2-7	F2-8.5	F2-10	F3-7	F3-8.5	F3-10
Grape pomace extract (mL)	210	210	210	210	210	210	210	210	210
Stevia (g)	12.5	12.5	12.5	12.5	12.5	12.5	12.5	12.5	12.5
Gelatin + water (gelatin hydration)	7 g + 14 mL	8.5 g + 17 mL	10 g + 20 mL	7 g + 14 mL	8.5 g + 17 mL	10 g + 20 mL	7 g + 14 mL	8.5 g + 17 mL	10 g + 20 mL

F1 – grape pomace extract with granularity of <125 µm; F2 – grape pomace extract with granularity of ≥125 – <200 µm; F3 – grape pomace extract with granularity of ≥200 – <300 µm; 7 – 7 g of gelatin; 8.5 – 8.5 g of gelatin; 10 – 10 g of gelatin.

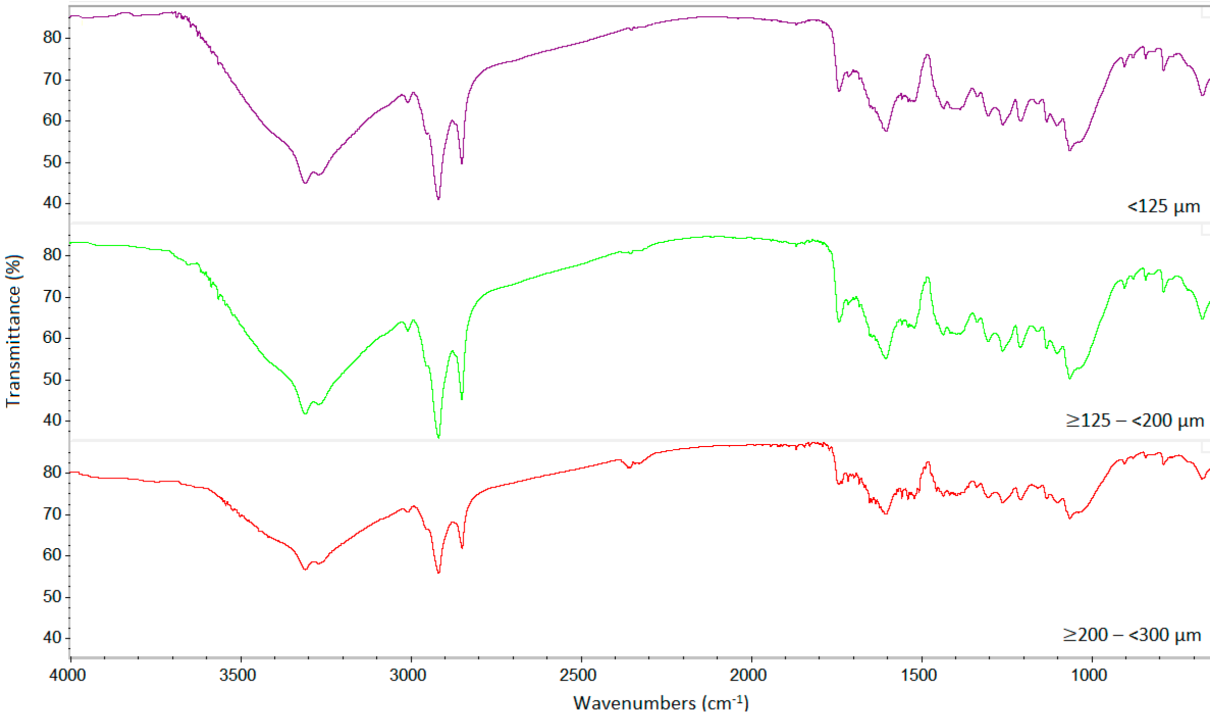

Figure S1. FT-IR spectra of Băbească Neagră grape pomace under the influence of different granularity
